# Risk perception, attitude, and practice related to COVID-19: A cross-sectional study among 1085 Iranian healthcare workers

**DOI:** 10.1016/j.amsu.2021.102865

**Published:** 2021-09-20

**Authors:** Romina Roshanshad, Amirhossein Roshanshad, Hossein Molavi Vardanjani, Amirali Mashhadiagha, Maryam Mobarakabadi, Armin Hoveidaei, Amir Human Hoveidaei

**Affiliations:** a Student Research Committee, Shiraz University of Medical Sciences, Shiraz, Iran; b MPH Department, Shiraz University of Medical Sciences, Shiraz, Iran; c Research Center for Traditional Medicine and History of Medicine, Shiraz University of Medical Sciences, Shiraz, Iran; d Health Policy Research Center, Shiraz University of Medical Sciences, Shiraz, Iran; e Neurophysiology Lab, Hamadan University of Medical Sciences, Hamadan, Iran; f Student Research Committee, School of Allied Medical Science, Shahid Beheshti University of Medical Sciences, Tehran, Iran; g Students' Scientific Research Center, Tehran University of Medical Sciences, Tehran, Iran

**Keywords:** Attitude, Healthcare workers, Health policy, Perceived stress scale, Practice, Risk perception

## Abstract

**Backgrounds:**

Healthcare workers are at risk of mental illness during COVID-19 pandemic. We investigated the level of perceived risk and adherence to preventive behaviors regarding COVID-19 among medical students and physicians.

**Materials and methods:**

We conducted an online nationwide cross-sectional survey among Iranian physicians and medical students. We collected data regarding perceived risk, perceived stress scale (P·S·S.), attitude, practice, and information sources. We assessed the association of four main outcomes (risk perception, P·S.S. score, attitude, and practice) with demographic variables.

**Results:**

A total of 1085 participants with an overall fear score of 6.48 ± 2.29 out of 10 filled the questionnaires. Older and more educated participants had a higher risk perception level and adherence to protective measures. In contrary, participants in lower-risk workplaces had lower risk perception scores. Main sources of information did not affect the scores of risk perception, P·S·S., and practice. Higher risk perception scores were observed in those with higher practice and P·S.S. scores.

Contrary to risk perception, the P·S.S. score was not affected by many of the demographic variables, except gender. The most positive attitude was observed in individuals with a higher level of trust in governmental information sources. Participants with higher P·S.S. scores adhered more to protective measures.

**Conclusion:**

Risk perception had the greatest impact on adherence to preventive behaviors. Therefore, policymakers should consider perceived risk as a predictor of the extension of the pandemic. Both risk perception and P·S.S. reflect the severity of fear from COVID-19; however, P·S.S. is less affected by sociodemographic and workplace characteristics.

## Highlights

• Risk perception had the greatest impact on the adherence to preventive behaviors.
• Risk perception and perceived stress both reflect severity of fear from COVID-19.
• P·S.S. is less affected by sociodemographic and workplace characteristics.
• Using a source of information would not affect the attitude of the H·C·W.s.
• Trust in a specific source of information might affect the attitude of the H·C·W.s.

## 1 Introduction

The COVID-19 pandemic has been the most challenging health problem since December 2019. Despite the progress in COVID-19 management, its death toll is still catastrophic [1]. According to the World Health Organization (Update: July 22, 2021), near 192 million people have caught the disease, and around 4.1 million deaths were reported due to the COVID-19 [2].

Healthcare workers (H·C·W.s) are at the frontline of the battle against COVID-19; they have a higher risk of contracting the infection rather than the general population [3]. Furthermore, due to the heavy workload, H·C·W.s suffer from excessive burnout in this unpredictable situation and are vulnerable to physical and mental illnesses [[4], [5], [6]]. Previous studies have shown that the pandemic impacted the health workers' mental health status and increased both mood and anxiety disorders [[7], [8], [9], [10]]. A negative relationship was observed between mental health and the perceived risk of H·C·W.s [11].

Based on the health behavior theories, the most motivated people to implement protective behaviors perceive the highest risk concerning the issue [12]. Accordingly, risk perception level may play a fundamental role in determining the chance of getting infected.

The media and the information that we take can influence our risk perception widely. In today's world, numerous information sources can have paradoxical effects on human behaviors. While it can facilitate the informing processes, it might be an uncontrollable route of tabloid and misinformation [12,13].

The gradual decrease in obeying preventive behaviors has led to the next peaks of COVID-19 cases in many countries, including Iran [14]; therefore, reassessing the rate of adherence to preventive behaviors and the level of the perceived risk of H·C·W.s is vital to control the outbreak especially in healthcare settings [15]. This study investigated the level of perceived risk and stress, level of trust to various available sources of information, and preventive behaviors adherence among Iranian medical students and practitioners regarding COVID-19.

## 2 Methods

### 2.1 Design

We conducted an online cross-sectional nationwide study during the second peak of COVID-19 in Iran when almost all of the COVID-19 referral centers had been overloaded. The research protocol has been registered in ResearchRegistery.com with the identifying number researchregistry6994 [16]. This work has been reported in line with the STROCSS criteria [17].

### 2.2 Study population and sample

Physicians and medical students were the study target population. Participants were sampled in four strata, including students within their first three years of the medical studentship (preclinical students: basic science students, without exposure to clinical care settings), students within their last four years of the studentship (clinical students: clerkship students and interns, with exposure to clinical care settings) and residents, general practitioners, and specialists. Details on medical education in Iran are presented elsewhere [18].

We applied Cochran's formula to estimate a proportion for sample size calculation [19], assuming a 0.05 type I statistical error, a 30% prevalence of having an adequate perceived risk among H·C·W.s, and a 10% of precision. The minimum sample size was calculated to be around 900. The minimum sample size was divided by a 40% response rate, estimating that near 2200 questionnaires should be sent to achieve the predetermined sample size.

### 2.3 Measurement tools

We collected data using a 41-item online self-administered questionnaire consisted of closed questions grouped in following parts: 1) background and demographic, 2) perceived risk, 3) perceived stress, 4) attitude toward ongoing measures for prevention and control the pandemic, 5) adherence to preventive measures and recommendation, 6) the most trusted information sources, 7) misinformation, and 8) perceived fear of pandemic.

Age, gender, strata (preclinical students, clerkship students or interns, residents, general practitioners, and specialists), city of education or practice (less than 0.5 million, between 0.5 and 1 million, more than 1 million population), risk of work-related exposure to COVID-19 (high, medium, or low-risk ward or place of working) were collected as demographic and background variables.

A questionnaire including nine questions, used in previous studies [20,21], was applied to assess the risk perception. Participants were asked to respond to each question on a 5-point Likert scale. Persian version of the Perceived Stress Scale (PSS-10) was used to assess the perceived stress; its reliability and validity have been proven before [22]. Three Likert scale questions were designed to measure participants' attitudes toward the performance of the H·C·W.s, hospitals and medical universities, and ministry of health, adopted from other studies [23,24]. To measure the extent of adherence to hygiene recommendations, seven Likert scale questions previously applied by Taghrir et al. [25] were adopted. The cumulative score of risk perception, attitude, and practice ranged from 9 to 45, 3 to 15, and 7 to 35, respectively. 13 different sources of information, including such as state T.V. channels, Newspapers, non-governmental news agencies, etc. were listed. Participants were asked to rank them considering trustfulness. Misinformation was assessed using two Likert scale questions: “COVID-19 is under control in Iran and its intensity is decreasing.” AND “The severity of COVID-19 is exaggerated and people are extremely concerned.” adapted from a study on misinformation about Ebola [24]. We used a visual analog scale to measure the level of fear about the Covid-19 pandemic. The items of the questionnaire are provided in the Supplementary file.

### 2.4 Data collection

The questionnaires were distributed online via two of the most widely used social media applications in Iran.

### 2.5 Data preparation

In this study, we assessed the association of these four main outcomes (risk perception, P·S.S. score, attitude, and practice) with demographics and these additional variables.

To achieve this purpose, participants were divided into two groups based on each main outcome variable, and 4 mentioned additional variables (trust in governmental sources, trust in foreign sources, misinformation, and fear of COVID-19). Two groups were labeled as “higher” and “lower” based on the group discussion's cut-off.

The cumulative risk perception score >34 (35–45), PSS score >23 (24–40), attitude score >10 (11–15), and practice score >33 (34 and 35), were categorized into higher group. For other variables, ones with 3–5 score for governmental trust, 4 to 5 score for foreign trust, 5 to 10 score for misinformation, and 8 to 10 score for fear were labeled as higher groups.

### 2.6 Statistical analysis

Data were analyzed using SPSS version 16.0 (I·B.M., Armonk, NY, U.S.A.). Descriptive analysis was used to demonstrate demographic variables. For categorical variables, the frequencies and percentages were estimated. The proportion of participants with a higher level of the outcome variable and their 95% confidence intervals (CI) was estimated, assuming a binomial distribution. A chi-squared test was used to find the association between qualitative variables. Independent association of independent and outcome variables was investigated applying multivariable binary logistic regression. Variable selection for multivariable modeling was made based on the conceptual study framework and then a univariate p-value of less than 0.3. Crude and adjusted odds ratio (OR) and their 95% CI were estimated. A two-tailed (p-value < 0.05) was considered statistically significant.

### 2.7 Ethical considerations

Our study was conducted concerning the tenets of the declaration of Helsinki. Shiraz University of Medical Sciences' ethics committee approved this study (ethical code: IR.sums.med.rec.1399.205). After informing the participants about our study's goals, they voluntarily gave their informed consent to fill the questionnaires.

## 3 Results

A total of 2169 medical students and practitioners opened the questionnaire's link; 1648 (76%) began filling the questions, while 1085 (response rate = 50.0%) submitted the completed questionnaire. Their mean age was 30.85 ± 12.42 years. Among the respondents, 19.8% worked in high-risk wards, 46.3% in medium-risk wards, and 33.9% in low-risk wards. The overall fear score was 6.48 ± 2.29 out of 10.

Older participants and those with higher educational levels had a higher level of risk perception (P < 0.001), adherence to protective measures (P < 0.001), and more positive attitudes toward government and healthcare system performance (P < 0.001). Demographic and background data are shown in Table 1.

**Table 1:** Demographics of the participants and differences in major outcomes according to demographics.

Variables	N (%)			Highest	
		Risk perception Prevalence(95% C·I)	PSS Prevalence(95% C·I)	Attitude Prevalence(95% C·I)	Practice Prevalence(95% C·I)
**Age**		<0.001	0.603	<0.001	<0.001
18–23	285(26.5)	27.6(22.3, 33.4)	35.2(29.6, 41.1)	39.9(34.1, 45.8)	32(26.5, 37.9)
24–30	503(46.7)	39.4(35, 43.8)	36.3(32.1, 40.7)	34.5(30.4, 38.9)	30(26, 34.3)
>31	289(26.8)	52.8(46.6, 58.9)	39.1(33.4, 45.1)	52.9(46.8, 58.8)	49.3(43.3, 55.3)
**Gender**		0.011	<0.001	0.118	<0.001
Female	654(60.5)	43(39, 46.9)	43.3(39.4, 47.2)	38.9(35.2, 42.8)	42.6(38.7, 46.5)
Male	427(39.5)	35.1(30.4, 39.9)	27.4(23.2, 31.9)	43.8(38.9, 48.7)	25.5(21.4, 30)
**Level of education**		<0.001	0.723	<0.001	<0.001
Preclinical students	155(14.5)	25.4(18.3, 33.5)	34.4(27, 42.5)	49(40.8, 57.3)	29.3(21.9, 37.6)
Clinical students and residents	567(52.9)	38.3(34.3, 42.5)	37.8(33.8, 42)	31.4(27.6, 35.4)	31.3(27.4, 35.3)
GP	185(17.3)	47.1(39.5, 54.8)	34.6(27.7, 42)	49.7(42.3, 57.2)	40.2(33, 47.8)
Specialist	165(15.4)	48.3(40.1, 56.6)	39(31.4, 47)	56.6(48.5, 64.4)	53.8(45.7, 61.6)
**Ward**		0.001	0.788	<0.001	0.774
High risk	207(19.8)	43.8(37, 51)	37.2(30.6, 44.2)	33.3(26.9, 40.2)	34(27.5, 40.9)
Medium risk	484(46.3)	43.6(39, 48.1)	37.7(33.4, 42.3)	34(29.8, 38.5)	36.8(32.5, 41.3)
Low risk	355(33.9)	31.4(26.4, 36.7)	35.4(30.4, 40.7)	54.5(49.2, 59.8)	36.3(31.1, 41.6)
**City population**		0.388	0.684	0.673	0.988
>1 million	717(67.1)	38.3(34.6, 42)	37.8(34.2, 41.4)	39.9(36.3, 43.6)	35.5(32, 39.2)
0.5–1 million	68(6.4)	46.3(34, 58.9)	32.8(21.8, 45.4)	44.8(32.6, 57.4)	36.4(24.9, 49.1)
<0.5 million	283(26.5)	40.7(34.9, 46.8)	36.2(30.6, 42.1)	41.8(36, 47.8)	35.9(30.2, 41.8)
**Sources of Information**		0.270	0.222	0.047	0.560
Governmental	246(22.8)	42.9(36.5, 49.5)	37.3(31.2, 43.8)	46.1(39.6, 52.6)	38.7(32.4, 45.2)
Foreign	371(34.3)	38.6(33.6, 43.9)	40.7(35.6, 45.9)	36.5(31.6, 41.7)	36.2(31.2, 41.4)
Relatives and Experts	227(21)	42.9(36.3, 49.8)	32.4(26.3, 39)	38.5(32.1, 45.2)	32.3(26.1, 38.9)
No specific source	237(21.9)	35.3(29, 42)	35.3(29.2, 41.8)	45.3(38.7, 51.9)	36.1(29.9–42.7)
**Trust in Information**					
Governmental information		0.003	0.004	<0.001	0.041
Most trust	426(39.5)	34.2(29.6, 39.1)	31.7(27.3, 36.4)	51.9(47, 56.8)	32(27.6, 36.8)
Foreign information		0.447	0.324	0.315	0.158
Most trust	436(41.1)	40.8(36.1, 45.7)	38.7(34.1, 43.5)	38.7(34, 43.4)	38.2(33.6, 43)
**Misinformation**		<0.001	0.005	0.002	0.004
Most Misinformation	133(12.4)	22.2(15.3, 30.5)	26(18.7, 34.3)	53.4(44.5, 62.2)	24.6(17.5, 32.9)
**Fear of COVID-19**		<0.001	<0.001	0.331	<0.001
Most Fear	373(34.6)	66.5(61.3, 71.4)	50.4(45.2, 55.6)	42.9(37.8, 48.1)	49.9(44.6, 55.1)

GP: general practitioner, P·S·S.: perceived stress scale.

Comparison of the wards revealed that participants in lower-risk workplaces had lower risk perception scores (P = 0.001) and more positive attitudes (P < 0.001). Workplace risk was not associated with P·S.S. and practice scores. Main sources of information did not affect the scores of risk perception, P·S·S., and practice.

**Fig. 1:**
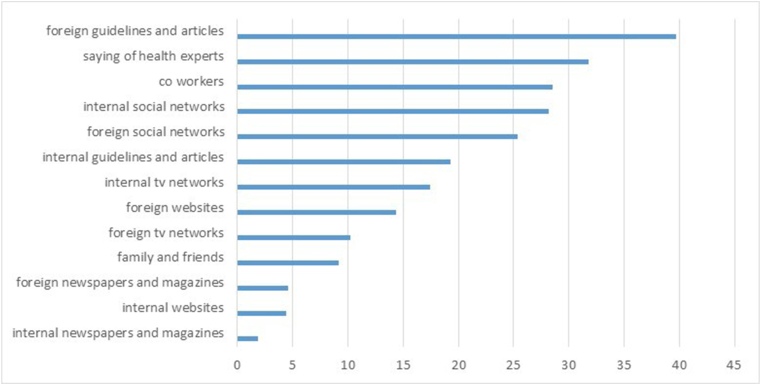
Sources of information that were used to obtain information about COVID-19.

Foreign guidelines and articles (39.7%), health expert comments (31.8%), and colleagues (28.5%) were the three main sources of information. Fig. 1 shows the frequency of the respondents' sources of information in detail, sorted from highest to lowest.

Higher risk perception scores were observed in those with higher practice (OR = 1.65, 95% CI: 1.21–2.24) and P·S.S. scores (OR = 1.84, 95% CI: 1.36–2.49), and ones who were working in the wards with higher exposure to COVID-19 (OR = 1.31, 95% CI: 1.06–1.61). In contrary to risk perception, the P·S.S. score was not affected by many of the demographic variables, except gender (male gender OR = 0.61, 95% CI: 0.46–0.82). Both of level of risk perception and practice affected PSS scores [(OR = 1.76, 95% CI: 1.31–2.36) and (OR = 1.29, 95% CI: 0.97–1.73), respectively]. Most positive attitude toward performance of the government and healthcare system was observed in individuals with more trust in governmental sources of information (OR = 2.16, 95% CI: 1.63–2.85) and those who adhered more to the protective measures (OR = 2.01, 95% CI: 1.51–2.69). Participants with higher P·S.S. scores adhered more to protective measures (OR = 1.31, 95% CI: 0.97–1.76). Table 2 demonstrates a univariate and multivariate analysis of risk perception, P·S·S., attitude, and practice.

**Table 2:** Univariate and multivariate analysis of risk perception, P·S·S., attitude, and practice.

Outcome variables	Independent variables	Crude OR(95% C·I)	Adjusted OR(95% C·I)
**Risk perception (higher vs. lower scores)**			
	Age	1.71(1.43, 2.05)	1.60(1.30, 1.98)
	Ward (ref. Low risk ward)	1.33(1.12, 1.59)	1.31(1.06, 1.61)
	Fear	5.82(4.40, 7.70)	5.06(3.73, 6.86)
	Misinformation	0.39(0.25, 0.60)	0.52(0.31, 0.86)
	Practice	2.39(1.84, 3.11)	1.65(1.21, 2.24)
	PSS	2.38(1.83, 3.09)	1.84(1.36, 2.49)
**P·S.S. (higher vs. lower scores)**			
	Male gender	0.49(0.38, 0.64)	0.61(0.46, 0.82)
	Fear	2.39(1.84, 3.10)	1.76(1.30, 2.38)
	Practice	1.62(1.25, 2.10)	1.29(0.97, 1.73)
	Risk perception	2.38(1.83, 3.09)	1.76(1.31, 2.36)
	Attitude	0.63(0.48, 0.81)	0.56(0.42, 0.74)
**Attitude (higher vs. lower scores)**			
	Male gender	1.22(0.95, 1.57)	1.33(1, 1.76)
	Education (ref. preclinical students)	1.3(1.13,1.49)	1.35(1.16, 1.58)
	Ward (ref. Low risk ward)	0.60(0.50, 0.72)	0.59(0.49, 0.71)
	Governmental Trust	2.09(1.63, 2.69)	2.16(1.63, 2.85)
	Misinformation	1.79(1.24–2.58)	1.64(1.09, 2.46)
	Practice	1.71(1.33–2.22)	2.01(1.51, 2.69)
	PSS	0.63(0.48–0.81)	0.61(0.46, 0.82)
**Practice (higher vs. lower scores)**			
	Male gender	0.46(0.35, 0.61)	0.48(0.36, 0.65)
	Education (ref. preclinical science students)	1.47(1.27, 1.69)	1.34(1.14, 1.58)
	Fear	2.52(1.93, 3.28)	1.6(1.17, 2.18)
	Misinformation	0.54(0.36, 0.83)	0.54(0.33, 0.87)
	PSS	1.62(1.25, 2.1)	1.31(0.97, 1.76)
	Risk perception	2.39(1.84, 3.11)	1.8(1.32, 2.45)
	Attitude	1.71(1.33, 2.22)	2.13(1.59, 2.86)

For these variables: risk perception, P·S·S., attitude, practice, fear, misinformation, trust in governmental and foreign sources, higher scores are compared with lower scores. For education and ward, preclinical students and low-risk wards are considered as the reference.

Details about participants' answers to each question are presented in the supplementary file.

## 4 Discussion

We showed older medical staff and ones who worked in high-risk wards had higher risk perception. Besides, risk perception, perceived stress, and adoption of preventive behaviors were all correlated with each other; an increase in each of these variables was accompanied by a concomitant rise in the two others. We also found that using a specific source of information cannot affect risk perception, perceived stress, practicing preventive behaviors, and a positive attitude toward healthcare system performance. It is actually the trust and belief in a source of information that may affect the participants' attitude.

In line with the previous studies [[26], [27], [28]], our survey results revealed that the participants who had higher risk perception and perceived stress demonstrated more adherence to preventive measures and recommendations. Wise et al. demonstrated that the adoption of preventive behaviors is mainly correlated with two aspects of risk perception assessment: the probability of getting infected personally and the perceived global impacts of COVID-19 [26]. Dryhurst et al. also found a similar correlation between risk perception and adoption of preventive behaviors in the general population of 10 European countries [29]. However, Taghrir et al. implied that preventive behaviors and risk perception were negatively correlated [25]. The observed difference in the results might be caused by the higher number of participants in the current study and the medical students' inclusion alongside the general practitioners and specialists. Compared to less educated and younger medical students, physicians with higher educational levels showed greater risk perception and subsequently more adherence to preventive behaviors.

Despite the concomitant changes in risk perception and perceived stress, they might have some subtle differences. Our results showed that while the risk perception was influenced by some environmental and demographic factors (e.g., age, risk of the workplace), perceived stress was only affected by gender. It seems that while both risk perception and perceived stress can reflect the amount of fear of a pandemic, perceived stress is mostly affected by an individual's baseline mental status. On the other hand, risk perception is correlated with environmental factors and exposure to the source of danger. This finding can be justified by the nature of the questions which were asked to assess each variable. PSS-10 questions assess the level of the stress of the respondents in the last month and do not focus on a special topic (in our case, COVID-19), while risk perception questions directly address COVID-19 and assess the current level of stress of the participants. Therefore, the relationship between perceived stress, risk perception, and workplace cannot be as simple as it may seem initially. One study on Tunisian physicians reported direct exposure to COVID-19 had no significant effect on their PSS-10 score [30]. However, a study in the U.S.A. reported more perceived stress (measured by the DASS-21 questionnaire) in physician trainees who had direct exposure compared to non-exposed trainees [31]. In another study in Oman, authors reported higher perceived stress levels (PSS-10) among H·C·W.s exposed to COVID-19 patients [32]. We divided our participants into three groups based on their workplace exposure to COVID-19: low, medium, and high-risk wards. This categorization enables a more precise and reliable comparison of the P·S.S. scores between these groups, as non-exposed groups in previous studies encompass a wide range of participants with different exposure to the infection. Besides, we included medical staff from the different educational levels of the whole country, yielding a more representative sample of the patients.

Our other interesting and noteworthy finding was that the source of information had no effect on any of the four measurements (P·S·S., risk perception, attitude, and practice), whereas participants' trust in governmental information sources had a significant impact on them. Our results showed that the higher trust in the governmental source of information was associated with a more positive attitude toward the healthcare system's performance. The more positive attitude was also correlated with better compliance with preventive behaviors. Therefore, we can hypothesize that the higher trust in information sources can indirectly lead to more adherence to hygiene protocols. A similar finding was reported in a survey in France; adopting preventive behaviors recommended by the authorities was directly associated with government trust [33]. This fact reminds the importance of authorities' role to provide trustworthy sources of information, leading to higher adherence to preventive behaviors and better control of the pandemic.

Despite the higher risk perception of medical staff who worked in high-risk wards, no significant differences in adopting preventive measures were observed between H·C·W.s of different wards. As H·C·W.s are mainly trained and educated individuals, they usually have appropriate access to more reliable sources of information, enabling them to consider the risk of infection even in lower-risk wards. Our results confirmed the mentioned hypothesis, as the most widely used sources of information in Iranian medical staff are foreign guidelines and articles, while social media was the main source of information among medical staff in other countries, e.g., India and U.A.E [[34], 35, [36]]. It shows that Iranian medical staff prefers to use more reliable and documented information sources, leading to an increase in their vigilance, even in lower-risk wards.

Sociodemographic characteristics may also affect risk perception. We found that older individuals and participants with higher educational levels had a higher level of risk perception, preventive behaviors, and a more positive attitude toward healthcare system performances. Jahangiry et al. also found similar findings in a sample of the general Iranian population: older and more educated individuals showed higher risk perception [37]. However, they realized that the perceived risk of susceptibility to COVID-19, and not the perceived severity of the disease, is higher in the elderly. Generally, older adults tend to have higher risk perception scores and less likely to engage in risky behaviors, especially in health-related issues [38]. Additionally, older people are at higher risk of severe infection [39], leading to greater perceived risk and engagement in preventive behaviors. The female gender also showed higher PSS-10 scores, which was consistent with a previous study in China [40]; therefore, they are expected to show a higher level of risk perception and more adherence to protective behaviors.

Despite the significant advances in developing different vaccines against COVID-19, social distancing and adherence to preventive behaviors remained an undeniable part of pandemic management. We demonstrated the impact of trust in information sources on risk perception, which is one of the most important determinants of adherence to preventive behaviors. Also, we compared the applicability of P·S.S. and risk perception scores for measurement of fear from the pandemic. These scores, alongside practice scores and trust in information sources, can be used to predict the extent of the spread of a pandemic. Estimating the extension of future pandemics during their early stages can be crucial as the nations can get prepared to manage the pandemics in advance. Then, we can minimize the catastrophic death tolls and the enormous burden of a pandemic.

This study came with some limitations. First, longitudinal evaluation of the participants was not possible, which is inherent to the cross-sectional studies. Besides, the distribution of the questionnaires via social media applications might come with a systematic bias. However, we distributed our questionnaire in almost all Iran provinces, resulting in a more representative and generalizable sample of the participants. Finally, we did not consider the participants' underlying mental health in the analysis, which might have impacted the P·S.S. and perceived risk or even practice scores.

## 5 Conclusion

Risk perception had the greatest impact on the adherence to preventive behaviors among Iranian H·C·W.s. Therefore, policymakers should consider perceived risk as a predictor of the extension of future pandemics. Then they can implement appropriate measures in advance before catastrophic events take place. We also found that while risk perception and P·S.S. both reflect the severity of fear from COVID-19, P·S.S. is less affected by sociodemographic and workplace characteristics. It is actually the trust in a specific source of information and not using the source of information by itself, which affects the attitude of the H·C·W.s.

## Provenance and peer review

Not commissioned, externally peer-reviewed.

## Ethics approval and consent to participate

Shiraz University of Medical Sciences' ethics committee approved this study (ethical code: IR.sums.med.rec.1399.205). After informing the participants about our study's goals, they voluntarily gave their informed consent to fill the questionnaires.

## Consent for publication

Not applicable.

## Availability of data and materials

Data is available from the corresponding author upon reasonable request via email.

## Funding

The project was financed by Vice Chancellor for Research of the 10.13039/501100005071Shiraz University of Medical Science (Grant No. 99-01-01-22617)

## Authors' contributions

A.R. designed the study and questionnaires, gathered, analyzed, and interpreted the data, and wrote the draft. R.R. gathered the data and wrote the draft. AM gathered the data and wrote the draft. A.H.H., A.H., and MM gathered the data and wrote the draft. H.M. helped in study design, data analysis, and revision. All authors read and approved the final manuscript.

## Declaration of competing interest

The authors declare that they have no competing interests.

## List of abbreviations

COVID-19: (coronavirus disease 2019)
P·S·S.: (perceived stress scale)
H·C·W.s: (healthcare workers)
CI: (confidence interval)
OR: (odds ratio)

## Annals of medicine and surgery

The following information is required for submission. Please note that failure to respond to these questions/statements will mean your submission will be returned. If you have nothing to declare in any of these categories then this should be stated.

## Registration of research studies

In accordance with the Declaration of Helsinki 2013, all research involving human participants has to be registered in a publicly accessible database. Please enter the name of the registry and the unique identifying number (UIN) of your study.

You can register any type of research at http://www.researchregistry.com to obtain your UIN if you have not already registered. This is mandatory for human studies only. Trials and certain observational research can also be registered elsewhere such as: ClinicalTrials.gov or ISRCTN or numerous other registries.

1. Name of the registry: http://www.researchregistry.com
2. Unique Identifying number or registration ID: researchregistry6994
3. Hyperlink to your specific registration (must be publicly accessible and will be checked):

https://www.researchregistry.com/register-now#home/registrationdetails/60fab790575d2e001efbfeb1/
