# Supplementary material for: Risk perception, attitude, and practice related to COVID-19: A cross-sectional study among 1085 Iranian healthcare workers
Source: Ann Med Surg (Lond). 2021 Sep 20;70:102865. doi: 10.1016/j.amsu.2021.102865 (PMC8450226; doi:10.1016/j.amsu.2021.102865)
Supplement: Multimedia component 2 [file mmc2.docx]

**Appendix**

Table S1. Practices of participants regarding COVID-19

| no | Item | Always  N (%) | Often  N (%) | Sometimes  N (%) | Rarely  N (%) | Never  N (%) |
| --- | --- | --- | --- | --- | --- | --- |
| 1 | I have postponed friendly appointments, going out and eating out and sports meetings | 51.5 | 24.3 | 14.1 | 7 | 3.1 |
| 2 | I have reduced the use of public transportation | 69.9 | 17.8 | 6.5 | 2.4 | 3.4 |
| 3 | I avoid going to public , closed spaces, and crowded places | 57.5 | 29.5 | 8.5 | 2.7 | 1.9 |
| 4 | I wash my hands more than before | 75.5 | 19.3 | 2.9 | 1.3 | 1 |
| 5 | I regularly disinfect surfaces that come in easy contact with the hands | 50.3 | 27 | 15.3 | 6 | 1.3 |
| 6 | I use personal protective equipment (especially masks) in the hospitals. | 80.8 | 14.7 | 3.2 | 0.6 | 0.8 |
| 7 | I use masks in the streets and in public places. | 71.3 | 19.5 | 6.1 | 1.7 | 1.3 |

Table S2. Misinformation about COVID-19

| no | Item | VERY HIGH  N (%) | HIGH  N (%) | MODERATE  N (%) | LOW  N (%) | VERY LOW  N (%) |
| --- | --- | --- | --- | --- | --- | --- |
| 1 | COVID-19 is under control in Iran and its intensity is decreasing. | 2 | 1.9 | 4.5 | 18.1 | 73.6 |
| 2 | The severity of COVID-19 is exaggerated and people are extremely concerned. | 2.2 | 2.9 | 7.4 | 24 | 63.5 |

Table S3. Attitude about COVID-19

| no | Item | VERY HIGH  N (%) | HIGH  N (%) | MODERATE  N (%) | LOW  N (%) | VERY LOW  N (%) |
| --- | --- | --- | --- | --- | --- | --- |
| 1 | The performance of the Ministry of Health and the National Headquarters for Combating Corona against COVID-19 has been appropriate. | 4.7 | 10.9 | 25.4 | 29.8 | 29.2 |
| 2 | Hospitals and universities have performed well against COVID-19 | 16.8 | 30.3 | 27 | 17.4 | 8.5 |
| 3 | The performance of the medical staff (doctors and nurses) against COVID-19 has been good. | 46 | 35.6 | 14.4 | 3 | 1 |

Table S4. Beliefs about COVID-19

| no | Item | VERY HIGH  N (%) | HIGH  N (%) | MODERATE  N (%) | LOW  N (%) | VERY LOW  N (%) |
| --- | --- | --- | --- | --- | --- | --- |
| **1** | How much do you trust governmental news and information? | 2.8 | 8.2 | 28.5 | 25.5 | 35 |
| **2** | How much do you trust foreign news and information? | 6.4 | 34.7 | 39.9 | 13.4 | 5.6 |

Table S5. Risk perceptions toward COVID-19

| NO | ITEM | very high  N (%) | high  N (%) | moderate  N (%) | low  N (%) | very low  N (%) |
| --- | --- | --- | --- | --- | --- | --- |
| 1 | If I get infected with COVID-19 , my health will be in great danger | 23.1 | 26 | 38.4 | 9.7 | 2.9 |
| 2 | I think COVID-19 is much more intense than influenza | 39 | 28.3 | 19.9 | 9.8 | 2.9 |
| 3 | COVID-19 has had a devastating effect on our society | 72.8 | 21.1 | 3.6 | 1.1 | 1.3 |
| 4 | I am more likely to get infected with COVID-19 than other people. | 28.1 | 28.5 | 24.7 | 12.7 | 6 |
| 5 | If my family and I have another medical problem, we will not go to the hospital because of Corona's risk. | 7.5 | 20.1 | 32.8 | 25.2 | 14.3 |
| 6 | I can take care of myself against COVID-19 | 16.7 | 42 | 30.6 | 7.8 | 2.9 |
| 7 | I'm worried about transmitting the disease to my family members. | 58.1 | 28.2 | 7.6 | 3.9 | 2.1 |
| 8 | I am worried about the protective equipment provided by the medical centers. | 48.4 | 26.2 | 16.4 | 6.4 | 2.6 |
| 9 | When I'm in a healthcare center, I still feel worry about my health even if I have full protective equipment. | 21 | 37.4 | 22.5 | 14 | 5.1 |

Table S6. PERCEIVED STRESS SCALE (PSS-10)

| NO | ITEM | VERY OFTEN  N (%) | Fairly Often  N (%) | Sometimes  N (%) | Almost Never  N (%) | NEVER  N (%) |
| --- | --- | --- | --- | --- | --- | --- |
| 1 | In the last month, how often have you been upset because of something that happened unexpectedly? | 33.2 | 26.5 | 24.5 | 10.9 | 4.9 |
| 2 | In the last month, how often have you felt that you were unable to control the important things in your life? | 20.1 | 22.4 | 27.6 | 20.8 | 9.2 |
| 3 | In the last month, how often have you felt nervous and “stressed”? | 26.5 | 29.5 | 24.9 | 14.6 | 4.4 |
| 4 | In the last month, how often have you felt confident about your ability to handle your personal problems? | 15.1 | 36.5 | 36.2 | 9.7 | 2.4 |
| 5 | In the last month, how often have you felt that things were going your way? | 5.3 | 20.6 | 41.3 | 23.7 | 9.1 |
| 6 | In the last month, how often have you found that you could not cope with all the things that you had to do? | 10.6 | 19.1 | 36.5 | 26.3 | 7.5 |
| 7 | In the last month, how often have you been able to control irritations in your life? | 7.3 | 28.9 | 44.4 | 15.2 | 4.1 |
| 8 | In the last month, how often have you felt that you were on top of things? | 6.6 | 27.3 | 46 | 15.8 | 4.3 |
| 9 | In the last month, how often have you been angered because of things that were outside of your control? | 9.3 | 16.4 | 31.9 | 32.1 | 10.3 |
| 10 | In the last month, how often have you felt difficulties were piling up so high that you could not overcome them? | 17.8 | 26.5 | 29.5 | 20.8 | 5.4 |

Table S7. Detailed sources of informaton

|  |  | N (%) |
| --- | --- | --- |
| governmental | TV networks | 189(17.4) |
|  | Newspapers and magazines | 21(1.9) |
|  | Social networks | 306(28.2) |
|  | Guidelines and articles | 209(19.3) |
|  | websites | 48(4.4) |
| foreign | TV networks | 111(10.2) |
|  | Newspapers and magazines | 50(4.6) |
|  | Social networks | 276(25.4) |
|  | Guidelines and articles | 431(39.7) |
|  | websites | 156(14.4) |
| Relatives and Experts | Family and friends | 100(9.2) |
|  | Co-workers | 309(28.5) |
|  | Saying of health experts | 345(31.8) |

Figure S1. The reasons for people disobedience to protective measures
